# Health promoting potential of herbal teas and tinctures from *Artemisia campestris* subsp. *maritima*: from traditional remedies to prospective products

**DOI:** 10.1038/s41598-018-23038-6

**Published:** 2018-03-16

**Authors:** Catarina Guerreiro Pereira, Luísa Barreira, Sebastiaan Bijttebier, Luc Pieters, Cátia Marques, Tamára F. Santos, Maria João Rodrigues, João Varela, Luísa Custódio

**Affiliations:** 1 Centre of Marine Sciences, University of Algarve, Faculty of Sciences and Technology, Ed. 7, Campus of Gambelas, 8005-139 Faro, Portugal; 2 University of Antwerp, Natural Products & Food Research and Analysis (NatuRA), Antwerp, Belgium; 3 Flemish Institute for Technological Research (VITO), Business Unit Separation and Conversion Technology (SCT), Mol, Belgium

## Abstract

This work explored the biotechnological potential of the medicinal halophyte *Artemisia campestris* subsp. *maritima* (dune wormwood) as a source of health promoting commodities. For that purpose, infusions, decoctions and tinctures were prepared from roots and aerial-organs and evaluated for *in vitro* antioxidant, anti-diabetic and tyrosinase-inhibitory potential, and also for polyphenolic and mineral contents and toxicity. The dune wormwood extracts had high polyphenolic content and several phenolics were identified by ultra-high performance liquid chromatography–photodiode array–mass-spectrometry (UHPLC-PDA-MS). The main compounds were quinic, chlorogenic and caffeic acids, coumarin sulfates and dicaffeoylquinic acids; several of the identified phytoconstituents are here firstly reported in this *A. campestris* subspecies. Results obtained with this plant’s extracts point to nutritional applications as mineral supplementary source, safe for human consumption, as suggested by the moderate to low toxicity of the extracts towards mammalian cell lines. The dune wormwood extracts had in general high antioxidant activity and also the capacity to inhibit α-glucosidase and tyrosinase. In summary, dune wormwood extracts are a significant source of polyphenolic and mineral constituents, antioxidants and α-glucosidase and tyrosinase inhibitors, and thus, relevant for different commercial segments like the pharmaceutical, cosmetic and/or food industries.

## Introduction

Medicinal plants are increasingly explored by the food industry for their health-promoting benefits either as readily available for herbal teas (e.g. *Matricaria chamomilla* [chamomile], *Cymbopogon citratus* [lemongrass]) or as sources of additives for functional foods and drinks (e.g. *Aloe vera* [aloe], *Aspalathus linearis* [rooibos])^1,2^. Yet, medicinal halophytes remain largely unexplored and underutilized despite their outstanding potential as a reservoir of bioactive compounds and innovative health promoting products^3^. Recently, different scientific efforts have unveiled some of these halophytes’ prospective commercial uses namely as food (e.g. *Arthrocnemum macrostachyum*^4^), herbal functional beverages (e.g. *Helichrysum italicum* subsp. *picardii*^5^, *Crithmum maritimum*^6^, *Limonium algarvense*^7^), or as raw material for pharmaceutical and other related industries (e.g. *Lithrum salicaria*^8^, *Polygonum maritimum*^9^).

Halophytes live and thrive in saline biotopes characterized by highly fluctuating abiotic constraints. To deal with such unfavourable conditions these salt-tolerant plants developed adaptive responses including the synthesis of highly bioactive molecules with potent antioxidant capacity, such as phenolic compounds, terpenoids and vitamins, to counteract reactive oxygen species (ROS) production and accumulation, inhibit oxidative chain-reactions and protect cellular structures^3^. These natural antioxidants usually display strong biological activities, like radical-scavenging, metal-chelating and enzyme-inhibiting abilities, leading to beneficial therapeutic properties, which can help explain the use of some halophytes in traditional medicine and as dietary plants^3,10^. For example, the aromatic *Crithmum maritimum* is used in folk medicine as diuretic, antiscorbutic, digestive or anti-inflammatory, and is traditionally consumed as condiment, pickle, and in salads^11^. Another aromatic halophyte, *Helichrysum italicum* subsp. *picardii*, is often used as a spice and has folk therapeutic uses such as anti-inflammatory, analgesic or anti-microbial^12^. Besides their traditional use as food and folk remedies, halophytes can be produced in otherwise uncultivable saline soils and marine-influenced environments and serve as alternative cash crops in saline agriculture. In fact, these plants could be explored for diverse commercial segments, from human and animal nutrition to pharmaceutical and cosmetic industries^13,14^.

*Artemisia campestris* L. subsp. *maritima* Arcangeli (Asteraceae), commonly named dune wormwood (“madorneira” or “erva-lombrigueira” in Portugal), is an aromatic and medicinal halophytic shrub common in coastal sand dunes throughout the temperate European Atlantic coast^15,16^. Usually consumed as herbal tea made from stems and leaves, it is described as a remedy to treat gastric disorders, hypertension and rheumatics, being also used for its anthelmintic and abortifacient properties^15^. The species, *A. campestris*, has additional ethnomedicinal uses described such as anti-diabetic, anti-inflammatory and antipyretic^16^. Although several studies have already profiled the phytochemical content and bioactivities of *A. campestris* (revised in Dib *et al*.^16^), only few reports focused on the subspecies *A. campestris* subsp. *maritima*. Research on this particular plant reports compounds like phenolic acids, flavonoids, coumarins, sesquiterpenes and acetophenone derivatives, determined on organic extracts^17–20^, and describes the antioxidant and anti-microbial activities of methanolic extracts^20^.

In folk medicine, water (infusions and decoctions) and hydro-alcoholic (tinctures) extracts are commonly used to convey the plants’ healing properties^21^. Considering the potential health benefits of such botanical extracts, medicinal plants can offer a wide range of bioactive components (e.g. polyphenols) and can be explored as raw material for herbal beverages, foods products or constituents in health promoting commodities^1^. In fact, natural products are currently in high demand and substances with anti-ageing or beauty-enhancement properties (e.g. skin whitening) are on top of consumers list of interest^1^. Other sought beneficial outcomes include management of diabetes mellitus and improvement of cognitive functions, associated with the intake of antioxidants^22^. Biochemical studies on medicinal plants can therefore be extremely useful to identify new sources of relevant products for pharmaceutical, cosmetic and/or food industries, and many *Artemisia* species already find extensive uses as food additives and in perfumery^23^. In this sense, *Artemisia campestris* subsp. *maritima* could be a potential reservoir of bioactive compounds, representing a commercial underexplored opportunity. Therefore, this work’s goal was to explore the dune wormwood’s biotechnological potential as source of bioactive phytochemicals. For that purpose, infusions, decoctions and tinctures were prepared from above and below-ground organs of *A. campestris* subsp. *maritima* and assessed for polyphenolic and mineral contents, and *in vitro* antioxidant, anti-diabetic and tyrosinase-inhibition potential. A preliminary *in vitro* toxicological assessment was also carried out using mammalian cells. To the best of our knowledge, this is the first time that such an attempt is made with this plant.

## Results and Discussion

### Phytochemical profile

The polyphenolic content of the extracts was firstly assessed in terms of their total contents of phenolics (TPC), flavonoids (TFC), condensed tannins (CTC), hydroxycinnamic acid derivatives (HAD), flavonols and anthocyanins (Table 1). Phenolic compounds are some of plants most widely occurring secondary metabolites^24^. Although there is no instituted classification in terms of high/low values of total phenolics, some authors state that natural extracts can be considered rich in phenolic compounds when their TPC is higher than 20 mg GAE/g DW^8,25,26^. In this sense, all of *A. campestris* subsp. *maritima* extracts have high phenolics content considering that TPC was between 114 and 134 mg GAE/g DW, with the highest value determined in aerial-organs’ tincture. This extract also had the highest flavonoid content (40.8 mg RE/g DW), higher HAD together with aerial-organs’ infusion and decoction (89.4–88.4 mg CAE/g DW), and higher anthocyanins along with roots’ tincture (3.46 and 3.36 mg CCE/g DW). Flavonols, on the other hand, were highest in roots’ tincture (66.2 mg QE/g DW). As for tannins content, it was not found in the dune wormwood samples (below the limit of quantification, which was 0.78 mg/g DW). Working with the same sub-species, Megdiche-Ksouri *et al*.^20^ reported similar total phenolics (159 mg GAE/g DW) but higher flavonoid (175 mg CE/g DW) and tannin (8.7 mg CE/g DW) contents in methanolic extracts from shoots. These differences could be ascribed not only to the different solvent and extraction procedure, which several studies have showed to greatly influence results, but also to the different analytical methods used^13^. In similar aqueous and hydro-alcoholic extracts from the aerial parts of the species *A. campestris*, other authors determined different level of TPC and TFC, either higher, similar or lower than those presently found^27–33^. These discrepant phytochemical contents may be explained by species-specific factors, harvesting time and/or environmental characteristics, since these variables affect the biosynthesis of secondary metabolites in plants^3,13^. Nevertheless, authors generally consider *A. campestris* rich in phenolic compounds^16,30^.

**Table 1:** Phenolic contents (mg/g dry weight, DW) of infusions, decoctions and tinctures from *Artemisia campestris* subsp. *maritima* organs and respective yields (infusion and decoctions: mg extract/200 mL, tinctures: mg extract/mL).

Organ	Extract	Yield	TPC^1^	TFC^2^	CTC^3^	HAD^4^	Flavonols^5^	Anthocyanins^6^
Roots	Infusion	100.9	115 ± 5.03^b^	25.0 ± 0.85^c^	<LQ	86.0 ± 0.64^bc^	59.5 ± 2.35^b^	1.90 ± 0.26^b^
	Decoction	142.0	114 ± 3.72^b^	26.3 ± 1.56^c^	<LQ	86.7 ± 0.98^bc^	57.8 ± 2.50^b^	2.44 ± 1.15^ab^
	Tincture	12.4	118 ± 5.23^b^	18.7 ± 0.98^d^	<LQ	85.0 ± 0.78^c^	66.2 ± 1.63^a^	3.36 ± 0.34^a^
Aerial-organs	Infusion	205.3	121 ± 6.20^b^	35.5 ± 0.69^b^	<LQ	89.2 ± 1.66^a^	54.1 ± 1.62^c^	1.96 ± 0.51^b^
	Decoction	208.5	119 ± 6.16^b^	34.4 ± 0.92^b^	<LQ	88.4 ± 1.96^ab^	51.9 ± 1.46^c^	1.94 ± 0.63^b^
	Tincture	20.1	134 ± 11.9^a^	40.8 ± 1.80^a^	<LQ	89.4 ± 1.83^a^	60.9 ± 0.94^b^	3.46 ± 0.53^a^

Data represent the mean ± SD (*n* ≥ 6). In each column, different letters mean significant differences (*p* < 0.05). LQ (limit of quantification) CTC = 0.78 mg CE/g DW.

^1^TPC: total polyphenol content, mg GAE/g DW, GAE: gallic acid equivalents.

^2^TFC total flavonoid content; mg QE/g DW, QE: quercetin equivalents.

^3^CTC: condensed tannin content, mg CE/g DW, CE: catechin equivalents.

^4^HAD hydroxycinnamic acid derivatives, mg CAE/g DW, CAE: caffeic acid equivalent.

^5^mg QE/g DW, QE: quercetin equivalents.

^6^mg CCE/g DW, CCE: cyanidin chloride equivalents.

**Figure 1:**
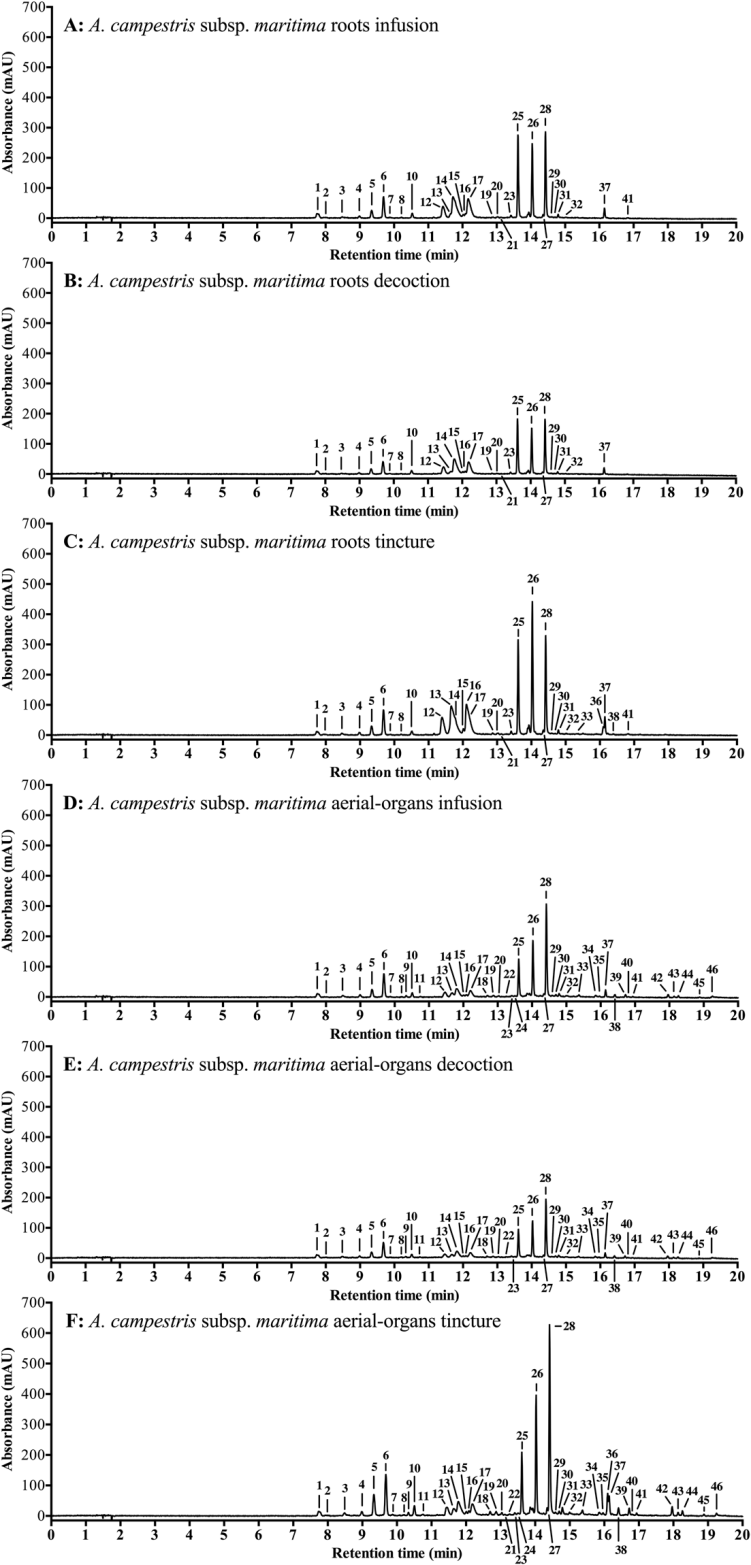
PDA chromatograms (280 + 330 nm) of the extracts from *A. campestris subsp. maritima* roots (**A**) infusion, (**B**) decoction, (**C**) tincture) and aerial-organs (**D**) infusion, (**E**): decoction, (**F**) tincture). Peak numbers refer to compounds listed in Tables 2 and 3.

To further explore the phytochemical profile of infusions, decoctions and tinctures from *A. campestris* subsp. *maritima* a generic LC-PDA-MS (liquid chromatography – photodiode array – mass spectrometry) method for moderately polar phytochemicals was employed. The analytical methodology was adapted from De Paepe *et al*.^34^, previously validated by those authors for quantitation of phenolic constituents in apple cultivars, and is fully detailed in Pereira *et al*.^5^ including performance characteristics, quantification procedures and compound tentative identification specifics. The aim was to (tentatively) identify phytochemical constituents in the dune wormwood extracts, getting an estimate of their concentrations and/or relative abundances when no reference standards were available. The phenolics and respective concentrations are presented in Table 2. As some standards can be expensive or not available, tentative identification of other compounds was accomplished based on available chromatographic and spectral information (Table 3). To get clean product ion spectra of the detected analytes, data dependent fragmentation was used. Product ions are substructures of precursor ions (ions of a particular mass over charge-range [*m/z*-range]), formed during fragmentation: structures were assigned to unknown peaks when both the *m*/*z*-values and molecular formulae/structures of the precursor and product ions were in agreement. Further information for de-replication was obtained from PDA spectra, in-house and commercial compound databases (PubChem^35^, Dictionary of Natural Products^36^, ChemSpider^37^) and peer reviewed publications (a more detailed explanation is given in Pereira *et al*.^5^). MS and diagnostic chromatographic data used for compound identification plus literature used for confirmation of compound identity can be found in Table S2 (supplementary material). It is important to mention that during LC-MS analysis different compounds can have different ionization efficiencies and so no absolute quantitative comparison can be made, although relative abundances per compound in-between samples can be calculated (based on the area of their most abundant ion). In this sense, the “maximum area detected” provides semi-quantitative information of compound abundance. Table 3 shows the relative abundances of these tentatively identified constituents. To visualize the extracts’ main detected compounds, the UV-chromatograms at combined wavelengths (280–330 nm, the absorption maxima of phenolics) are represented in Fig. 1, despite not showing all the constituents identified (compounds with no assigned peaks had low abundances or possibly their peaks overlapped).

**Table 2:** Concentrations of compounds in infusions, decoctions and tinctures from *Artemisia campestris* subsp. *maritima* organs (mg/g DW), calculated with reference standards using LC-amMS. Quantitation limits are presented as ≤ LOQs (µg/mg DW).

^a^Peak n°	Compound (Peak)	^b^RT (min)	Roots			Aerial-organs		
			Infusion	Decoction	Tincture	Infusion	Decoction	Tincture
	Quinic acid	1.52	13.00	14.00	15.00	24.00	24.00	24.00
	Protocatechuic acid	7.17	0.100	0.090	0.110	0.420	0.430	0.270
	*p*-Hydroxybenzoic acid	9.55	0.015	≤0.021	0.020	0.087	0.095	0.116
6	Chlorogenic acid	9.72	8.400	9.000	10.00	11.00	10.00	16.00
	4-Hydroxybenzaldehyde	10.27	0.007	0.010	0.017	0.009	0.009	0.018
	Syringic acid	10.32	0.049	≤0.062	0.081	≤0.048	≤0.066	0.047
10	Caffeic acid	10.56	0.920	0.970	0.630	0.920	1.000	1.630
18	Rutin	12.73	0.024	0.021	0.038	0.700	0.740	1.300
	Cynaroside	12.82	≤0.019	≤0.029	≤0.017	0.029	0.034	0.044
19	Coumaric acid	12.93	0.075	0.064	0.100	0.170	0.190	0.330
21	Ferulic acid	13.04	0.070	0.064	0.078	0.034	0.032	0.055
22	Isoquercitrin	13.29	0.021	0.029	0.024	0.120	0.130	0.200
24	Taxifolin	13.53	≤0.047	≤0.071	≤0.042	0.066	≤0.076	0.092
29	Salicylic acid	14.55	0.062	0.049	0.092	0.120	0.120	0.190
41	Luteolin	16.97	0.021	≤0.028	0.022	0.190	0.200	0.470
	Quercetin	17.15	≤0.005	≤0.007	≤0.004	0.052	0.080	0.061
	Naringenin	17.49	≤0.049	≤0.073	≤0.044	≤0.057	≤0.078	0.053
	Apigenin	18.50	≤0.005	≤0.007	≤0.004	0.016	0.016	0.034
	Isorhamnetin	18.57	≤0.048	≤0.073	0.044	0.160	0.200	0.250
	Kaempferol	18.85	0.020	0.030	0.018	0.026	0.036	0.024
		**TOTAL**	**22.78**	**24.33**	**26.27**	**38.12**	**37.31**	**45.18**

^a^Corresponding peak number in the chromatograms on Fig. 1. ^b^RT – retention times.

**Table 3:** Average relative abundances (peak area/mg DW, %) of the tentatively identified compounds in extracts from *Artemisia campestris* subsp. *maritima* organs, analysed by LC-PDA-amMS.

^a^Peak n°	Tentative ID	^b^RT (min)	Roots			Aerial-organs			Maximum area detected
			Infusion	Decoction	Tincture	Infusion	Decoction	Tincture	
1	Chlorogenic acid isomer (isochlorogenic acid A, B or C)	7.77	91	100	62	91	100	88	410 976 815
2	Hydroxybenzoic acid isomer (2,3-Dihydroxybenzaldehyde)	8	99	100	98	66	77	79	169 361 809
3	Hexoside of scopoletin (scopolin)	8.48	30	28	43	73	66	100	185 080 059
4	Hexoside of coumarin with 2 methoxy moieties (iso-fraxidin or fraxidin)	9.02	43	46	65	69	64	100	403 174 862
5	Chlorogenic acid isomer (isochlorogenic acid A, B or C)	9.33	48	50	49	55	56	100	1 097 618 207
	Aesculetin	9.61	19	19	44	43	47	100	68 226 925
7	Chlorogenic acid isomer (isochlorogenic acid A, B or C)	9.88	65	61	68	68	65	100	108 537 786
8	Fraxetin	10.19	100	93	82	60	62	87	82 132 702
9	Coumaric acid hexoside isomer	10.32	4	3	5	63	64	100	180 996 161
11	Coumaric acid hexoside isomer	10.76	6	4	7	69	70	100	102 579 661
12	Coumarin sulfate with 2 methoxy moieties (iso-fraxidin or fraxidin)	11.54	71	68	100	55	55	69	9 544 319 167
13	Coumarin sulfate (fraxetin-*O*-sulfate isomer)	11.65	50	53	31	82	86	100	4 395 956 598
	Not identified (C_12_H_18_O_7_S)	11.74	6	4	9	62	63	100	1 434 495 637
14	Coumarin sulfate (fraxetin-*O*-sulfate isomer)	11.78	75	73	100	34	35	41	23 258 593 384
15	Scopoletin	12.03	68	62	100	46	49	72	231 549 513
16	Coumarin with 2 methoxy moieties (iso-fraxidin or fraxidin)	12.11	93	90	95	64	64	100	256 857 167
17	Coumarin sulfate (scopoletin-*O*-sulfate isomer)	12.19	71	67	100	31	32	41	17 799 164 012
20	Fraxidin-caffeoyl-hexoside	13.03	32	30	45	55	55	100	91 320 194
23	Methoxy-cinnamic acid	13.53	46	52	100	30	34	55	22 190 655
25	Dicaffeoylquinic acid	13.65	92	100	91	59	62	69	1 324 940 207
26	Dicaffeoylquinic acid	14.06	75	77	100	68	64	93	1 473 103 666
27	Dicaffeoylquinic acid methyl ester	14.35	71	62	100	53	47	95	11 010 820
28	Dicaffeoylquinic acid	14.45	63	66	64	79	75	100	2 500 658 869
30	Dicaffeoylquinic acid methyl ester	14.6	34	34	46	67	76	100	24 968 283
31	Dicaffeoylquinic acid	14.79	44	44	55	52	48	100	190 893 063
32	Dicaffeoylquinic acid methyl ester	15.03	20	19	23	69	70	100	35 407 749
33	Dicaffeoylquinic acid methyl ester	15.37	9	8	11	52	47	100	136 311 796
34	Caffeic acid coupled to C_11_H_12_O_6_	15.89	6	3	3	97	100	100	206 701 996
35	Flavonoid	15.99	3	2	3	59	60	100	207 351 776
36	Ethoxy or dimethoxycinnamic acid	16.1	0	0	43	0	0	100	3 704 845 930
37	Tricaffeoylquinic acid	16.13	14	13	23	11	11	100	793 875 076
38	Dimethoxyflavonoid (axillarin)	16.41	2	1	3	48	51	100	635 257 416
39	Methoxyflavonoid (tamarixetin, rhamnetin, eupafolin, quercetin-3-methylether)	16.72	2	2	3	54	55	100	806 869 736
40	Methoxyflavonoid (laricitrin or mearnsetin)	16.8	0	1	1	61	100	86	89 733 523
	Trimethoxyflavonoid	17.49	2	1	2	48	49	100	40 757 681
42	Dimethoxyflavonoid (cirsiliol)	17.97	2	1	3	53	56	100	714 900 053
43	Trimethoxyflavonoid	18.13	2	1	3	53	55	100	171 818 097
44	Methoxyflavonoid (hispidulin)	18.26	2	1	3	53	56	100	738 797 835
45	Trimethoxyflavonoid (cirsilineol or eupatorin)	18.89	1	1	2	44	45	100	53 729 586
46	Tetramethoxyflavonoid	19.25	2	1	3	49	48	100	138 813 684
	Trimethoxyflavonoid (cirsilineol or eupatorin)	19.48	3	2	4	49	49	100	40 802 811
	Dimethoxyflavonoid (cirsimaritin)	20.07	2	0	2	41	42	100	17 338 075
	Linderoflavone B	21.2	NF	NF	NF	37	47	100	591 906

NF – not found.

^a^Corresponding peak number in the chromatograms on Fig. 1.

^b^RT – retention times.

According to Table 2, the dune wormwood aerial-organs’ extracts had greater diversity and higher levels of practically all phenolics found. Aerial-organs’ tincture in particular had higher concentrations of most of the determined compounds adding up to a total of 45 µg/mg DW. From this total, quinic acid amounts to half (24 µg/mg DW), followed by chlorogenic (16 µg/mg DW) and caffeic (1.6 µg/mg DW) acids. In fact, these phenolic acids were the main constituents determined in all extracts particularly quinic (roots: 13–15 µg/mg DW, aerial-organs: 24 µg/mg DW) and chlorogenic (roots: 8.4–10 µg/mg DW, aerial-organs: 10–16 µg/mg DW) acids, both higher in tinctures. Rutin was also preferentially detected in aerial-organs aqueous and hydro-alcoholic samples (0.7–1.3 µg/mg DW), followed by protocatechuic acid (0.27–0.43 µg/mg DW), luteolin (0.19–0.47 µg/mg DW) and coumaric acid (0.17–0.33 µg/mg DW), along with isoquercitrin, isorhamnetin and salicylic acid (~0.1–0.2 µg/mg DW). In roots’ extracts, protocatechuic acid in all extracts (0.09–0.11 µg/mg dw), and coumaric and salicylic acids in tincture (0.1 and 0.09 µg/mg dw, respectively) were also found in higher levels, although in comparatively lower concentrations than in the aerial-organ’s extracts. In Table 3 and Fig. 1 it is also possible to observe the higher compound diversity in extracts from aerial-organs, especially tinctures. However, relative abundance of some major constituents such as coumarin sulfates (peaks 12, 14 and 17) and dicaffeoylquinic acids (peaks 25 and 26) was higher in roots’ extracts, particularly tincture. Aerial-organs’ extracts had higher amounts of another coumarin sulfate (peak 13) and dicaffeoylquinic acid (peak 28), along with a chlorogenic acid isomer (peak 5) and an ethoxy/dimetoxycinnamic acid (peak 36). Again, it should be stated that Table 3 provides relative quantitative measures of abundance, not to be interpreted as absolute quantitative comparison. Overall, tinctures of both organs showed higher abundance and diversity of constituents comparatively to aqueous extracts and, between organs, extracts from aerial-organs had greater variety of phenolics, generally in higher levels. To the best of our knowledge, this is the first report comparing anatomical organs in this *Artemisia* species. Megdiche-Ksouri *et al*.^20^ also report a wide assortment of phytochemicals in dune wormwood’s shoots, several of them also presently determined, but studies detailing compound abundance in *A. campestris* extracts other than essential oils are extremely scarce. In fact, only Jahid *et al*.^33^ reports levels of phenolics in leaves’ hydro-alcoholic extracts with the main components catechin and vanillic acid (>20 mg/g DW), not being found in the current study, syringic (6 mg/g DW) and coumaric (0.9 mg/g DW) acids, presently determined at lower concentrations (0.05–0.08 mg/g DW and 0.06–0.33 mg/g DW, respectively), and caffeic acid (0.2 mg/g DW), being one of the current main constituents particularly in aerial-organs’ tincture (1.6 mg/g dw). These authors^33^ also consider that compound nature and abundance are related to environmental conditions, a well-established notion when comparing intra-species phytochemical content^3,13,38,39^.

Nevertheless, and although differing considerably between subspecies^38^, the phenolic profiles of *A. campestris* compiled in literature are generally in agreement with that reported here and include compounds like phenolic acids such as caffeic, chlorogenic, isochlorogenic and other dicaffeoylquinic acids, flavonoids such as apigenin, rutin, luteolin, kaempferol and quercetin, or hydroxycoumarins like aesculetin and scopoletin^16,19,20,40–42^. In fact, from the wide variety of phenolic constituents (tentatively) identified in *A. campestris* subsp. *maritima* extracts (Tables 2 and 3), most if not all were already described in the *Artemisia* genus. However, for the species *A. campestris* no reports were found detailing quinic, protocatechuic, *p*-hydroxybenzoic and salicylic acids, 4-hydroxybenzaldehyde, cynaroside, isoquercitrin and taxifolin (although its derivatives are described), which are, to the best of our knowledge, here described for the first time in the species. Moreover, chlorogenic, syringic, caffeic, coumaric and ferulic acids, luteolin, apigenin and kaempferol were not found reported in the literature for the subspecies under study (although derivatives for the three later are reported) and are therefore here firstly described in *A. campestris* subsp. *maritima*.

### Mineral composition

Aqueous extracts like herbal teas can be considered an added source of minerals for the human diet^2,6^. In this context, the presence of these essential nutrients in the dune wormwood’s extracts could be of added value for their potential use as food products or in herbal beverages. Hence, *A. campestris* subsp. *maritima* extracts were analysed for mineral content and Table 4 summarizes the results. The most abundant element was Na (9.10–32.6 mg/g DW), followed by K (3.32–15.6 mg/g DW) and Ca (0.09–4.53 mg/g DW), all in higher levels in aerial-organs aqueous extracts. Magnesium (Mg: 0.39–1.67 mg/g DW) and Fe (22–1059 µg/g DW) were also relatively abundant but with higher levels in roots aqueous extract. Mn and Zn were determined in lower concentrations (Mn: 3.31–87.9 µg/g DW; and Zn: 2.30–18.3 µg/g DW). Mn was more abundant in aerial-organs aqueous samples and Zn had similar levels on aqueous extracts of both above and below-ground organs. Moreover, tinctures had consistently lower mineral content showing that water extracts are better at extracting these nutrients from the plant. In fact, herbal teas are usually considered good sources of many elements such as Na, Ca, K, Mg, Fe, Mn or Zn^2^. Considering the adult daily dietary reference mineral intakes (Na: 1200–1500, Ca: 1000–1300, K: 4700–5100, Mg: 255–350, Fe: 5.0–23, Mn: 1.8–2.6 and Zn: 6.8–10.9 mg/day^43^), one gram of the dune wormwood’s aqueous extracts could supply up 5% of Mn and 21% of Fe (with regard to the minimum reference values), without reaching the maximum recommended daily intake of Na, and therefore may contribute to the adult daily intakes of some major and minor elements. Moreover, values of Cu and Cr can be considered low and safe for consumption as they are below the recommended dietary allowance values (Cu: 700–1000, Cr: 20–45 µg/day)^43^ and potentially toxic minerals like Ni, Cd and Pb were not detected (below the LOQs). Even if these were present in the extracts at undetected levels, they would not constitute a threat since the LOQs, when adjusted to the equivalent units based on the extraction yields (Table 1), are below legislated values for plants (Pb 0.3 μg/g and Cd 0.2 μg/g of plant material; EC Regulation 1881/2006). Overall, results highlight a possible nutritional role of the dune wormwood’s extracts, particularly aerial-organs and aqueous extracts, as an additional mineral source.

**Table 4:** Mineral content (mg or μg/g DW) in extracts of infusions, decoctions and tinctures from *Artemisia campestris* subsp. *maritima* organs.

	Mineral	Roots			Aerial-organs		
		Infusion	Decoction	Tincture	Infusion	Decoction	Tincture
	Macro-elements (mg/g)						
Essential elements	Na	17.5 ± 1.79^bc^	19.4 ± 1.43^b^	9.10 ± 0.65^d^	32.6 ± 1.79^a^	32.4 ± 1.19^a^	12.3 ± 1.88^cd^
	Ca	2.91 ± 0.02^b^	2.99 ± 0.11^b^	0.09 ± 0.01^c^	4.53 ± 0.05^a^	4.29 ± 0.19^a^	0.15 ± 0.01^c^
	K	11.0 ± 1.55^b^	11.4 ± 1.63^ab^	3.32 ± 0.33^c^	15.6 ± 1.49^a^	14.5 ± 0.20^ab^	3.67 ± 0.37^c^
	Mg	1.67 ± 0.12^a^	1.65 ± 0.06^a^	0.52 ± 0.08^c^	1.32 ± 0.05^b^	1.16 ± 0.06^b^	0.39 ± 0.03^c^
	Micro and trace-elements (μg/g)						
	Fe	1059 ± 105^a^	926 ± 57.1^a^	<LOQ	630 ± 98.7^b^	626 ± 22.5^b^	22.0 ± 0.82^c^
	Mn	76.8 ± 2.72^ab^	70.7 ± 3.15^b^	3.31 ± 1.97^c^	87.9 ± 6.77^a^	79.9 ± 3.59^ab^	3.75 ± 0.31^c^
	Zn	16.2 ± 1.88^a^	18.3 ± 0.55^a^	<LOQ	17.4 ± 2.88^a^	18.0 ± 2.33^a^	2.30 ± 0.85^b^
	Cu	27.2 ± 0.93^ab^	31.5 ± 5.08^a^	6.70 ± 0.00^c^	14.6 ± 0.44^bc^	13.2 ± 1.42^bc^	1.81 ± 0.90^c^
	Cr	0.54 ± 0.01^a^	0.79 ± 0.03^a^	0.32 ± 0.00^a^	0.79 ± 0.06^a^	0.78 ± 0.05^a^	0.11 ± 0.00^a^
	Ni	<LOQ	<LOQ	<LOQ	<LOQ	<LOQ	<LOQ
Non-essential elements	Pb	<LOQ	<LOQ	<LOQ	<LOQ	<LOQ	<LOQ
	Cd	<LOQ	<LOQ	<LOQ	<LOQ	<LOQ	<LOQ

Data represent the mean ± SD (*n* = 3). In each row different letters mean significant differences (*p*<0.05).

LOQs: Fe: 0.48 μg/g, Zn: 0.88 μg/g, Ni: 0.31 μg/g, Pb: 0.71 μg/g, Cd: 0.40 μg/g of extract DW.

### Toxicological evaluation

**Figure 2:**
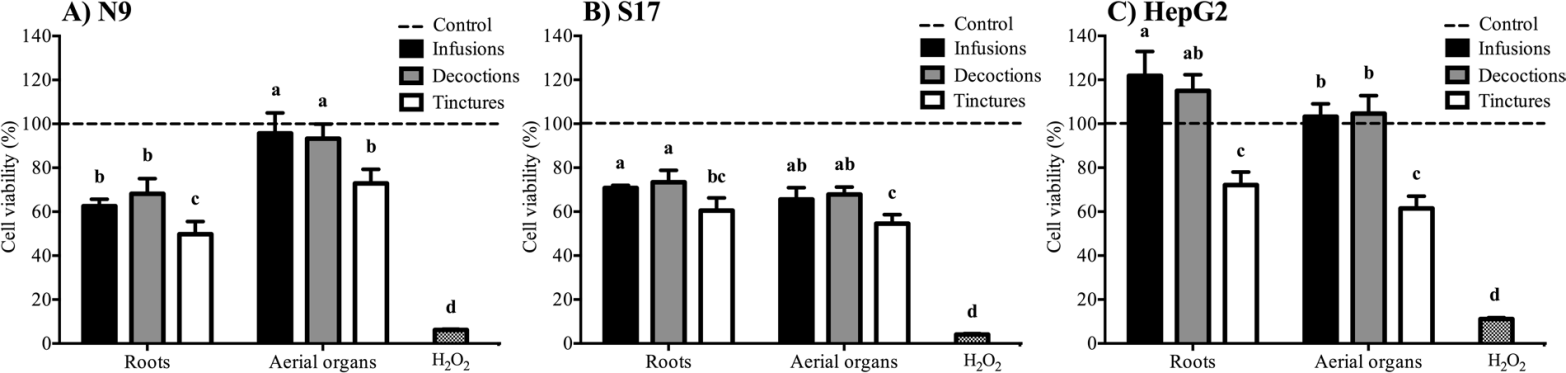
Toxicity of infusions, decoctions and tinctures (100 μg/mL extract dw) from *Artemisia campestris* subsp. *maritima* organs on mammalian cell lines: (**A**) N9, (**B**) S17 and (**C**) HepG2. Cells treated only with cell culture medium were used as controls; H_2_O_2_ was used as positive control for cell toxicity. Values represent the mean ± SD of at least three experiments performed in triplicate (n = 9). In each graph, different letters mean significant differences (*p* < 0.05).

The potential toxicity of new herbal products for human use, such as plant extracts, must be determined to establish its safe consumption. Preliminary toxicological evaluations can be made by *in vitro* models that address the sensitivity of mammalian cell lines to possible toxic effects of the extracts, delivering reliable and quick results and reducing *in vivo* testing^5–7,44,45^. Aiming at such a predictive toxicity screening, the dune wormwood extracts were tested for cytotoxicity towards three mammalian cell lines and the resulting cellular viabilities are presented in Fig. 2. The aqueous extracts showed overall low toxicity with cell viability values higher than those obtained for tinctures. Infusions and decoctions exerted no toxic effects in the hepatocarcinoma (HepG2) cells while tinctures had moderate to low toxicity with cellular viabilities between 62% (aerial-organs) and 72% (roots). For the microglia (N9) cell line, toxicity of the aerial-organs aqueous extracts was very low (>90% viabilities) while that of aerial-organs tincture (73% viability) and roots infusion and decoction (63–68% viabilities) can be considered moderate to low; roots’ tincture exerted a more toxic effect with 50% of cellular viability. For the stromal (S17) cells, roots’ aqueous extracts had low toxicity (71–73% viabilities) whereas roots’ tincture (61% viability) and aerial-organs water extracts (66–68% viabilities) were only moderately toxic; aerial-organs’ tincture resulted in 55% of cellular viability. As a preliminary safety evaluation of *A. campestris* subsp. *maritima* extracts, results suggest that they may be regarded as safe for consumption, although some caution is advised regarding the use of hydro-alcoholic extracts. Nevertheless, for comparison purposes, the widely consumed green tea had cellular viabilities as low as 30% in S17 cells^7^. Moreover, acute toxicity tests of *A. campestris* leaves aqueous extracts on mice showed that up to 3200 mg/kg body weight administered orally neither killed nor impaired behaviour^42^ and intraperitoneal injections rendered a LD_50_ equivalent to 2500 mg/kg b.w.^28^.

### Biological activities

Antioxidants can be considered a group of medicinally preventive molecules also used as food additives to inhibit food oxidation. Hence, natural antioxidant sources are increasingly sought after as an alternative to synthetic antioxidants in the food, cosmetic and therapeutic industries^3,22^. Antioxidants are scavengers of free radicals or ROS and deactivators of metal catalysts by chelation, among other activities, reducing oxidative stress and consequent cell damage. It is increasingly documented that dietary antioxidant phytochemicals effectively prevent oxidative damage, reducing the risk of oxidative-stress related conditions like neurodegenerative and vascular diseases, carcinogenesis or inflammation^10,22,46^. Their intake is also associated with the management of diabetes mellitus^22^ and amelioration of skin ageing conditions^47^.

In this work, the antioxidant potential of the dune wormwood’s extracts was assessed by eight different methods targeting radical scavenging activity (RSA) and metal-related potential (Table 5). The extracts were overall effective as scavengers of DPPH, ABTS, NO and O_2_^•—^ radicals and at reducing iron, but their chelating properties were moderate for copper and low for iron. In the DPPH assay the aerial-organs’ tincture had the lowest IC_50_ value (240 µg/mL), lower than that obtained for the positive control (BHT; IC_50_ = 320 µg/mL), followed by aerial-organs’ infusion (330 µg/mL), decoction (340 µg/mL) and roots decoction (370 µg/mL), all similar to BHT (*p* < 0.05). High RSA against DPPH was also reported by Megdiche-Ksouri *et al*.^20^ in methanolic extracts from shoots of the same *A. campestris* subspecies. Aerial-organs’ tincture also had the strongest NO scavenging activity allowing an IC_50_ of 290 µg/mL, comparable to that of this organs’ decoction (490 µg/mL, *p* < 0.05); most interestingly all extracts were better NO scavengers than the positive control (ascorbic acid, IC_50_ = 2.31 mg/mL). This was also the case with O_2_^•—^ scavenging as catechin had the highest IC_50_ (620 µg/mL). For this radical’s assay, however, the lowest IC_50_ value was obtained after the application of roots’ decoction (180 µg/mL), followed by infusions from both organs (roots: 210 µg/mL, aerial-organs: 230 µg/mL). Roots decoction was also the best ABTS scavenger (IC_50_ = 370 µg/mL), statistically similar to the result obtained with the aerial-organs’ tincture (IC_50_ = 400 µg/mL; *p* < 0.05). As for the iron reducing capacity, the best result was obtained with the aerial-organs’ infusion with an IC_50_ of 170 µg/mL, followed by aerial-organs’ tincture (230 µg/mL), roots tincture (240 µg/mL) and decoction (250 µg/mL). This is in accordance with Megdiche-Ksouri *et al*.^20^ findings of a high FRAP in this subspecies. Conversely, the extracts iron-chelating activity was comparatively low, with IC_50_ values higher that 5 mg/mL, while the capacity to chelate copper was moderate (best IC_50_ = 1.3 mg/mL in aerial-organs’ water extracts). Tannins were not detected in any of the extracts, which may partially explain its low chelating potential since tannins are known metal chelating agents^48^. The aerial-organ’s water extracts had the highest capacity to chelate both metals (CCA, IC_50_ = 1.30–1.31 mg/mL; ICA, IC_50_ = 6.33–6.47 mg/mL). Several studies previously highlighted the high antioxidant capacity of similar aqueous and hydro-alcoholic extracts from *A. campestris*^27,29,30,32,33,42^, which confirms our results of strong *in vitro* antioxidant potential for this subspecies. Most of these authors also credited the pronounced antioxidant activity of the extracts to the polyphenolic content which is, in fact, an association widely reported by several studies that confirm the phenolics’ role as antioxidants, especially in halophyte plants^3^. Accordingly, aerial-organs’ tincture had the highest levels of almost all phenolics groups (Table 1) and was also of the best-scoring extracts in terms of antioxidant activity. Actually, that extract also had overall higher abundance and variety of individual phenolic constituents (Tables 2 and 3), altogether corroborating the hypothesis that phenolics play a major role in the sample’s strong antioxidant potential. For example, the main components quinic, chlorogenic and caffeic acids, determined in higher amounts in aerial-organs’ tincture (Table 2), are known antioxidant compounds^49–51^. Nevertheless, roots’ extracts showed greater relative abundances of some major constituents (Table 3), such as the dicaffeoylquinic acid (peak 25, Fig. 1) in roots’ decoction, and quinic, chlorogenic and caffeic acids, although in lower levels than in aerial-organs’ samples, were the predominant constituents. Synergistic and/or additive effects between these phytoconstituents may also account for the equally high antioxidant activity of roots’ decoction.

**Table 5:** Antioxidant activity (IC_50_ values, mg/mL) of infusions, decoctions and tinctures from *Artemisia campestris* subsp. *maritima* organs: radical scavenging on DPPH, ABTS, NO and O_2_^•—^ radicals, ferric reducing antioxidant power (FRAP) and metal-chelating activities on copper (CCA) and iron (ICA).

Samples			Antioxidant activity						
	Organ	Extract	DPPH	ABTS	NO	O_2_^•—^	FRAP	CCA	ICA
*A. campestris* subsp. *maritima*	Roots	Infusion	0.39 ± 0.02^c^	0.45 ± 0.02^de^	0.74 ± 0.03^c^	0.21 ± 0.01^b^	0.29 ± 0.01^c^	1.64 ± 0.10^c^	7.82 ± 0.37^d^
		Decoction	0.37 ± 0.02^bc^	0.37 ± 0.01^b^	0.55 ± 0.01^bc^	0.18 ± 0.01^a^	0.25 ± 0.00^b^	1.64 ± 0.04^c^	7.37 ± 0.34^cd^
		Tincture	0.46 ± 0.02^d^	0.46 ± 0.01^e^	1.40 ± 0.07^d^	0.33 ± 0.01^d^	0.24 ± 0.00^b^	3.60 ± 0.11^e^	>10
	Aerial-organs	Infusion	0.33 ± 0.03^b^	0.41 ± 0.01^cd^	0.70 ± 0.03^bc^	0.23 ± 0.01^bc^	0.17 ± 0.00^a^	1.31 ± 0.05^b^	6.47 ± 0.35^bc^
		Decoction	0.34 ± 0.03^bc^	0.44 ± 0.01^de^	0.49 ± 0.01^ab^	0.24 ± 0.00^c^	0.27 ± 0.01^c^	1.30 ± 0.09^b^	6.33 ± 0.43^b^
		Tincture	0.24 ± 0.01^a^	0.40 ± 0.01^bc^	0.29 ± 0.02^a^	0.35 ± 0.01^e^	0.23 ± 0.01^b^	2.51 ± 0.09^d^	>10
BHT*			0.32 ± 0.02^b^	0.11 ± 0.00^a^			—		
Ascorbic acid*					2.31 ± 0.22^e^				
Catechin*						0.62 ± 0.01^f^			
EDTA*								0.13 ± 0.00^a^	0.07 ± 0.00^a^

Values represent the mean ± SD of at least three experiments performed in triplicate (n = 9). In each column different letters mean significant differences (p < 0.05). *Positive controls.

Besides antioxidant activity, other bioactivities have been ascribed to extracts from *A. campestris* as for example hypoglycaemic effects^28^. Type 2 diabetes mellitus (T2DM) is a common health disorder characterized by high blood glucose levels that can lead to major metabolic complications if left untreated^52^. One effective strategy to manage T2DM is to inhibit carbohydrate-hydrolysing enzymes, such as α-glucosidase, delaying carbohydrate digestion and uptake and resulting in reduced postprandial blood glucose levels, therefore lowering hyperglycaemia linked to T2DM^52,53^. In this sense, the dune wormwood’s extracts were tested for their capacity to inhibit microbial and mammalian α-glucosidases as an assessment of their anti-diabetic potential.

All extracts had the ability to inhibit the microbial α-glucosidase but the most active samples were roots’ aqueous extracts and aerial-organs’ decoction (IC_50_ = 0.89–1.13 mg/mL). Interestingly, all of the extracts were more efficient at inhibiting the microbial α-glucosidase than the positive control used acarbose (IC_50_ = 3.14 mg/mL), a clinically used inhibitor of this enzyme. However, only the roots’ extracts were able to inhibit mammalian α-glucosidase, particularly roots’ tincture (IC_50_ = 2.90 mg/mL), still more active than acarbose (IC_50_ = 4.64 mg/mL). Roots’ extracts were less active towards the mammalian enzyme than for the microbial counterpart, an outcome already described for some compounds showing that enzyme origin can influence the extracts’ inhibition of α-glucosidase^54^. Nevertheless, and despite the notion that the mammalian enzyme is a more reliable proxy for *in vivo* activity^54^, the *in vivo* anti-diabetic potential of *A. campestris* aqueous extracts from leaves was demonstrated by Sefi *et al*.^28^, having significantly reduced blood glucose levels in diabetic rats. Those authors considered that the *in vivo* hypoglycaemic activity of *A. campestris* extracts could be related to its strong antioxidant properties, and stated the role that this plant’s water extracts can have on the treatment of diabetic patients^28^. It is recognized that polyphenolic compounds, besides potent antioxidants^3,10^, can also have glucosidase-modulating activities therefore contributing to the management of T2DM^52^. The dune wormwood’s extracts had a high phenolic content and contained some compounds with described hypoglycaemic activity, namely chlorogenic, caffeic and ferulic acids^50,51^, and with reported α-glucosidase inhibitory activity, like isoquercitrin, luteolin, quercetin and apigenin^52^. Overall, our results suggest that all dune wormwood’s extracts could be beneficial in managing T2DM by its capacity to inhibit dietary carbohydrate digestive enzymes, which was higher than acarbose, and consequently controlling glucose levels. Furthermore, as oxidative stress has been considered a mediator in diabetic complications^55^, the extracts’ strong antioxidant potential can also be an adjuvant in preventing or attenuating the disease’s symptoms when used in combined anti-diabetic strategies.

Skin hyperpigmentation (e.g. melasma, freckles, age spots) is a result of melanin over-production but, as tyrosinase is essential in melanin biosynthesis, inhibition of this enzyme can help prevent and/or manage undesired skin darkening^47,56^. Tyrosinase is also responsible for unwanted browning of fruits and vegetables, which decreases their market value^56,57^. Hence, tyrosinase inhibitors from natural sources are increasingly sought not only for cosmetic and medicinal purposes but also for their potential in improving food quality^47,56,57^. In this context, the tyrosinase inhibitory potential of the dune wormwood’s extracts was evaluated and results are depicted on Table 6. All extracts were active, particularly aerial-organs’ infusion (IC_50_ = 4.13 mg/mL), although less effective than the used positive control (arbutin, IC_50_ = 0.48 mg/mL). Tyrosinase is a copper-containing enzyme^56^ and thus the extracts’ moderate copper chelating activity could be related to their tyrosinase inhibitory capacity. In fact, metal chelating and ROS-scavenging properties are mechanisms often thought to be related with the reducing activity of flavonoids^47^. Some flavonoids were already identified as tyrosinase inhibitors, as for example quercetin, kaempferol and taxifolin, the last being as effective as arbutin^57^. All these compounds were detected in the dune wormwood’s extracts, possibly contributing to their tyrosinase inhibitory activity. To the best of our knowledge, this is the first report on the tyrosinase inhibitory potential of *A. campestris* subsp. *maritima*.

**Table 6:** Inhibitory activities (IC_50_ values, mg/mL) on microbial and mammalian α-glucosidase enzymes, and on tyrosinase enzyme of infusions, decoctions and tinctures from *A. campestris* subsp. *maritima* organs.

Samples	Organ	Extract	Microbial α-glucosidase	Mammalian α-glucosidase	Tyrosinase
*A. campestris* subsp. *maritima*	Roots	Infusion	0.92 ± 0.04^a^	6.09 ± 0.41^c^	7.58 ± 0.14^d^
		Decoction	0.89 ± 0.03^a^	6.62 ± 0.48^c^	5.56 ± 0.45^c^
		Tincture	2.54 ± 0.05^c^	2.90 ± 0.22^a^	5.23 ± 0.12^c^
	Aerial-organs	Infusion	1.64 ± 0.05^b^	>10	4.13 ± 0.27^b^
		Decoction	1.13 ± 0.03^a^	>10	5.14 ± 0.35^c^
		Tincture	1.62 ± 0.06^b^	>10	5.35 ± 0.25^c^
Acarbose*			3.14 ± 0.23^d^	4.64 ± 0.76^b^	
Arbutin*					0.48 ± 0.01^a^

Values represent the mean ± SD of at least three experiments performed in triplicate (n = 9). In each column different letters mean significant differences (*p* < 0.05). *Positive controls.

This study reports for the first time a comprehensive assessment of the biotechnological potential of *A. campestris* subsp. *maritima* as a source of innovative products with health promoting properties. Overall, our results point to the potential role of infusions, decoctions and tinctures of the dune wormwood in the prevention of oxidative-stress related diseases and in the management of diabetes and skin-hyperpigmentation conditions. More specifically, those formulations can be considered an unexplored source of polyphenolic and mineral constituents, antioxidants and α-glucosidase and tyrosinase inhibitors that could deliver raw material to different commercial segments including the pharmaceutical, cosmetic and/or food industries. Further studies are being pursued aiming to fully explore the health-promoting benefits of this plant’s extracts, namely their *in vivo* effects.

## Methods

### Plant collection

*Artemisia campestris* L. subsp*. maritima* Arcang. (Compositae) plants were collected in South Portugal, within the area of the Ria Formosa coastal lagoon, near Faro (Ludo, 37°2′6.526′′N 7°58′58.465′′W), in June of 2013. The taxonomical classification was carried out by Dr. Manuel J. Pinto, botanist in the National Museum of Natural History, University of Lisbon, Botanical Garden, Portugal, and a voucher specimen (voucher code MBH34) is kept in the herbarium of Marbiotech’s laboratory. Plants were divided in roots and aerial-organs (stems and leaves), oven dried at 50 °C until complete dryness (3 days), milled and stored at −20 °C until use.

### Extracts preparation: infusions, decoctions and tinctures

Water extracts were prepared similarly to a regular cup-of-tea: 1 g of dried plant material was homogenized in 200 mL of ultrapure water. For infusions, the biomass was immersed in boiling water for 5 min; for decoctions, the biomass was boiled in water for 5 min. Hydro-ethanolic extracts were prepared similarly to a home-made tincture: 20 g of dried plant material was left homogenising in 200 mL of 80% aqueous ethanol for a week. Independent extractions (n ≥ 3) for each combination of method + plant-part were made. All extracts were filtered (Whatman n° 4), vacuum and/or freeze-dried and stored in a dark, cool and moist-free environment. Extracts were re-suspended in water or aqueous ethanol to a concentration of 10 mg/mL to determine (spectrophotometric) phenolic content and test for bioactivities. For these assays, no significant differences were found among corresponding extracts from the different extractions and therefore freeze-dried extracts were pooled accordingly for the remaining analyses.

### Phytochemical composition of the extracts

#### Total polyphenols (TPC), flavonoids (TFC) and condensed tannin (CTC) content

The TPC, TFC and CTC were estimated by spectrophotometric methods, respectively: Folin-Ciocalteau, aluminium chloride colorimetric and 4-dimethylaminocinnamaldehyde (DMACA), as described in Rodrigues *et al*.^26^. Gallic acid, quercetin and catechin were used as standards and results are presented as milligrams of standard equivalents per gram of extract dry weight (GAE, QE and CE, respectively; mg/g dw). Further information pertained to these methods is presented in Table S1 (supplementary material).

#### Hydroxycinnamic acid derivatives (HAD), flavonols and anthocyanins content

Total contents in HAD, flavonols and anthocyanins were assessed spectrophotometrically as described previously^26^ using caffeic acid, quercetin and cyanidin chloride as standards, respectively. Results are presented as milligrams of standard equivalents per gram of extract dry weight (CAE, QE and CCE, respectively; mg/g dw). Further information pertained to these methods is presented in Table S1 (sup. material).

#### Profile of moderately polar compounds by UHPLC

Standard stock solutions were prepared at 1 mg/mL in UHPLC-grade methanol and stored at 4 °C in the dark. Standard dilutions were prepared in 60:40 (*v*:*v*) methanol:40 mM ammonium formate buffer (reference standards: apigenin, apigenin-7-O-glucoside (apigetrin), catechin, cyanidin-3-O-arabinoside, cyanidin-3-O-galactoside chloride (ideain chloride), cyanidin-3-O-glucoside chloride (kuromanin chloride), cyanidin-3-O-rutinoside chloride (keracyanin chloride), (+)-dihydrokaempferol ((+)-aromadendrin), epicatechin, epigallocatechin, epigallocatechin gallate, flavone, galangin, hesperidin, hesperidin methyl chalcone, 4-hydroxybenzaldehyde, kaempferol, kaempferol-3-O-glucoside (astragalin), limonin, luteolin, naringenin, naringin, neohesperidin dihydrochalcone, phloretin, phloretin-O-20-glucoside (phloridzin), procyanidin B2, protocatechuic acid, propyl gallate, quercetin, quercetin-3-O-arabinoside (avicularin), quercetin-3-O-galactoside (hyperin), quercetin-3-O-glucoside (isoquercitrin), quercetin-3-O-rhamnoside (quercitrin), rutin, uvaol, and caffeic, chlorogenic, coumaric, dihydrocaffeic, ellagic, ferulic, gallic, gentisic, m-hydroxybenzoic, hydroferulic, p-hydroxybenzoic, oleanolic, quinic, rosmarinic, salicylic, sinapinic and syringic acids). Freeze-dried pooled extracts (approx. 15 mg) were dissolved in 20 mL of 60:40 methanol:water +40 mM ammonium formate, followed by 1 min vortex mixing, 30 min sonication (40 kHz, 100 W, room temperature) and 10 min centrifugation (3000 rpm). Supernatants were diluted 100-fold and stored along with undiluted extracts at 4 °C, until analysis. Both undiluted and diluted extracts were analysed with a generic ultra-high performance liquid chromatography – photodiode array – accurate mass mass spectrometry (UHPLC-PDA-amMS) method for moderately polar phytochemicals adapted from De Paepe *et al*.^34^ and fully detailed in Pereira *et al*.^5^. Briefly, for analysis 5 µL of extract was injected on an UPLC BEH SHIELD RP18 column (3.0 mm × 150 mm, 1.7 µm; Waters, MA) and thermostatically eluted (40 °C) with a quaternary solvent manager and a ‘Hot Pocket’ column oven. The mobile phase consisted of water +0.1% formic acid (A) and acetonitrile +0.1% formic acid (B), following a gradient of (min/%A): 0.0/100, 9.91/74, 18.51/35, 18.76/0, 23.76/0, 23.88/100, 26.00/100. For detection, a Q Exactive MS (Thermo Fisher Scientific, Bremen, Germany) was used with heated electrospray ionization (HESI). For quantitative analysis, full scan data were acquired using polarity switching with a mass/charge (*m*/*z*) range of 120–1800 and resolving power set at 70 000 at full width at half maximum (FWHM). Data were also recorded using data dependent fragmentation (ddMS^2^) in positive and negative ionization mode to obtain additional structural information. The PDA detector was set to scan from 190 to 800 nm during all analyses. The lowest calibration point included in the calibration curve was used to calculate the limits of quantitation (LOQs). The concentration ranges described by De Paepe *et al*.^34^ were also used during the present work. Results regarding concentrations of identified compounds were calculated as µg/mg of extract dry weight.

### Mineral composition

Freeze-dried pooled extracts were digested in a combination of nitric acid (HNO_3_) and hydrogen peroxide on a hot plate and evaporated until dryness (up to 24 h). Digested samples were diluted in 20 mL of 5% HNO_3_ and analysed for mineral content by Microwave Plasma-Atomic Emission Spectrometer (MP-AES; Agilent 4200 MP-AES, Agilent Victoria, Australia), as described in Pereira *et al*.^6^. Instrumental detection limits were as follows: Ca: 0.04 μg/L, Cd: 1.4 μg/L, Cr: 0.3 μg/L, Cu: 0.5 μg/L, Fe: 1.7 μg/L, K: 0.6 μg/L, Mg: 0.031 mg/L, Mn: 0.1 μg/L, Na: 0.1 μg/L, Ni: 1.1 μg/L, Pb: 2.5 μg/L and Zn: 3.1 μg/L. Results were expressed as mg or μg/g of extract dry weight (DW). Appropriate blanks were also produced and analysed.

### Toxicological evaluation of the samples

Samples’ toxicity was assessed using murine microglia (N9), murine bone marrow stromal (S17) and human hepatocellular carcinoma (HepG2) cell lines. The N9 cell line was provided by the Faculty of Pharmacy and Centre for Neurosciences and Cell Biology (University of Coimbra, Portugal), S17 and HepG2 cells were delivered by the Centre for Biomedical Research (CBMR, University of Algarve, Portugal). Cell culture was maintained as described in Pereira *et al*.^6^. Toxicity was evaluated according to Rodrigues *et al*.^7^. Briefly, N9 cells where plated at an initial density of 1 × 0^4^ cells/well while S17 and HepG2 cells were seeded at 5 × 10^3^ cells/well, all in 96-well plates. Freeze-dried pooled extracts were dissolved in culture medium (100 μg/mL) and incubated with cells for 72 h; culture medium was used as negative control and hydrogen peroxide (H_2_O_2_) as positive control. Cell viability was determined by the MTT (3-(4,5-dimethylthiazol-2-yl)-2,5-diphenyltetrazolium bromide) assay and results were expressed in terms of cell viability (%).

### Biological activities

#### Antioxidant activity assessed by four radical-based assays

The extracts’ radical scavenging capacity against the DPPH (1,1-diphenyl-2picrylhydrazyl), ABTS (2,2′-azino-bis(3-ethylbenzothiazoline-6-sulfonic acid), NO (nitric oxide) and O_2_^•—^ (superoxide) radicals was assessed as described in Rodrigues *et al*.^7,26^. BHT (butylated hydroxytoluene), ascorbic acid and catechin were used as positive controls. Results were calculated as percentage of antioxidant activity in relation to a control containing ultrapure water or aqueous ethanol, and expressed as IC_50_ values (mg/mL; half maximal inhibitory concentration, ascertained for extracts with activities higher than 50% at 10 mg/mL).

#### Antioxidant activity assessed by three metal-related assays

The extracts’ chelating ability towards copper (CCA) and iron (ICA) and their Fe^3+^ reducing capacity (ferric reducing antioxidant power, FRAP) were assessed as described previously^26^. EDTA (ethylenediamine tetraacetic acid) and BHT were used as positive controls. Results were calculated as percentage of antioxidant activity relative to a positive control for FRAP, and in relation to a negative control (ultrapure water/aqueous ethanol) for CCA and ICA, and were expressed as IC_50_ values (mg/mL).

#### *In vitro* anti-diabetic activity: inhibition of microbial and mammalian α-glucosidases

The microbial α-glucosidase enzyme was obtained from the yeast *Saccharomyces cerevisiae*; rat’s intestine acetone powder was used to obtain a crude enzyme extract as an example of a mammalian-origin α-glucosidase. The extracts’ capacity to inhibit both enzymes was assessed following Kwon *et al*.^53^ and using acarbose as positive control. Results are expressed as IC_50_ values (mg/mL), calculated as percentage of inhibitory activity in relation to a control (ultrapure water/aqueous ethanol).

#### In vitro tyrosinase inhibition

The extracts’ ability to inhibit tyrosinase was assessed following Custódio *et al*.^58^, using arbutin as positive control. Results, calculated as percentage of inhibitory activity in relation to a control (ultrapure water/aqueous ethanol), are expressed as IC_50_ values (mg/mL).

### Statistical analysis

Experiments were conducted at least in triplicate and results were expressed as mean ± standard deviation (SD). Significant differences (*p* < 0.05) were assessed by one-way analysis of variance (ANOVA) followed by Tukey pairwise multiple comparison test or, when parametricity of data did not prevail, Kruskal Wallis one-way analysis of variance on ranks followed by Dunn’s test. Statistical analyses were executed using XLStat^®^ version 19.4. IC_50_ values were computed by curve fitting in GraphPad Prism^®^ version 6.0c.

### Data Availability

The datasets generated during and/or analyzed during the current study are available from the corresponding author on reasonable request.

## Electronic supplementary material

Supplementary Material
