# Supplementary material for: Health promoting potential of herbal teas and tinctures from Artemisia campestris subsp. maritima: from traditional remedies to prospective products
Source: Sci Rep. 2018 Mar 16;8:4689. doi: 10.1038/s41598-018-23038-6 (PMC5856739; doi:10.1038/s41598-018-23038-6)
Supplement: Supplementary file 1 — Supplementary Material [file 41598_2018_23038_MOESM1_ESM.docx]

**supplementary MATERIAL**

**Health promoting potential of herbal teas and tinctures from *Artemisia campestris* subsp. *maritima*: from traditional remedies to prospective products**

Catarina Guerreiro Pereira^a^, Luísa Barreira^a^, Sebastiaan Bijttebier^b,c^, Luc Pieters^b^, Cátia Marques^a^, Tamára F. Santos^a^, Maria João Rodrigues^a^, João Varela^a^, Luísa Custódio^a^*.

^a^Centre of Marine Sciences, University of Algarve, Faculty of Sciences and Technology, Ed. 7, Campus of Gambelas, 8005-139 Faro, Portugal.

^b^University of Antwerp, Natural Products & Food Research and Analysis (NatuRA), Antwerp, Belgium.

^c^Flemish Institute for Technological Research (VITO), Business Unit Separation and Conversion Technology (SCT), Mol, Belgium.

***Corresponding author:** Luísa Custódio, CCMAR, University of Algarve, Faculty of Sciences and Technology, Ed. 7, Campus of Gambelas, 8005-139 Faro, Portugal. Telephone: +351 289 800900 ext. 7381. E-mail: lcustodio@ualg.pt.

**Running Title: Bioactivities and chemical characterization of dune wormwood**

**Table S1.** Information pertained to the quantification of total polyphenol content (TPC), total flavonoid content (TFC) condensed tannin content (CTC), hydroxycinnamic acid derivatives (HAD), flavonols and anthocyanins: calibration curve equations, linearity, detection limits and limits of quantification.

|  | **CCE** | **Linearity** | **DL (mg/mL)** | **LOQ (mg/g DW)** |
| --- | --- | --- | --- | --- |
| **TPC** | y = 1.2800x + 0.1287 | R^2^ = 0.9882 | 0.006 | 2.067 ^1^ |
| **TFC** | y = 3.4394x + 0.1132 | R² = 0.9940 | 0.002 | 0.599 ^2^ |
| **CTC^3^** | y = 1.0453x + 0.1385 | R² = 0.9857 | 0.002 | 0.781 ^3^ |
| **HAD^4^** | y = 3.9839x + 0.3250 | R² = 0.9984 | 0.005 | 1.534 ^4^ |
| **Flavonols^5^** | y = 3.3706x + 0.1116 | R² = 0.9990 | 0.003 | 1.146 ^5^ |
| **Anthocyanins^6^** | y = 15813x + 0.0769 | R^2^ = 0.9808 | 0.001 | 0.316 ^6^ |

CCE: Calibration curve equation; DL: Detection limits; LOQ: Limit of quantification

^1^ mg GAE/g DW, GAE: gallic acid equivalents

^2^ mg QE/g DW, QE: quercetin equivalents

^3^ mg CE/g DW, CE: catechin equivalents

^4^ mg CAE/g DW, CAE: caffeic acid equivalent

^5^ mg QE/g DW, QE: quercetin equivalents

^6^ mg CCE/g DW, CCE: cyanidin chloride equivalents

**Table S2.** Chromatographic and spectral data of the tentatively identified compounds in infusions, decoctions and tinctures from *A. campestris* subsp. *maritima* organs, detected with a generic LC-PDA-amMS method for moderately polar phytochemicals.

| **Compound tentative ID** | **Molecular formula** | **HESI neg full MS** | **HESI neg ddMS^2^** | **HESI pos full MS** | **HESI pos ddMS^2^** | ***RT (min)** | **Max UV absorb. (nm)** | **Previously reported** |
| --- | --- | --- | --- | --- | --- | --- | --- | --- |
| **Chlorogenic acid isomer (isochlorogenic acid A, B or C)** | C_16_H_18_O_9_ | 353.08804 [M-H]^-^ | 191.1; 179.0; 173.0; 135.0 | 355.10217 [M+H]^+^; 377.08395 [M+Na]^+^ | 163.0; 135.0 | 7.77 | 300; 326 | [1][2] |
| **Hydroxybenzoic acid isomer (2,3-Dihydroxybenzaldehyde)** | C_7_H_6_O_3_ | 137.02452 [M-H]^-^ | 119.0; 109.0; 108.0; 93.0; 81.0 | 139.03917 [M+H]^+^ | 111.0; 93.0; 65.0 | 8 | - | - |
| **Hexoside of scopoletin (scopolin)** | C_16_H_18_O_9_ | 399.09436 [M-H+FA]^-^ | 191.0; 176.0; 148.02 | 372.12912 [M+NH_4_]^+^; 377.08454 [M+Na]^+^ | 193.0; 178.0; 165.0; 133.0 | 8.48 | 284; 339 | [2][3] |
| **Hexoside of coumarin with 2 methoxy moieties (iso-fraxidin or fraxidin)** | C_17_H_20_O_10_ | 429.10516 [M-H+FA]^-^; 383.09921 [M-H]^-^ | 221.0; 206.0; 191.0; 163.0 | 402.13951 [M+NH_4_]^+^; 407.09479 [M+Na]^+^; 791.20083 [2M+Na]^+^; 786.24508 [2M+NH_4_]^+^ | 223.1; 208.0; 190.0; 135.0 | 9.02 | 291; 327 | - |
| **Chlorogenic acid isomer (isochlorogenic acid A, B or C)** | C_16_H_18_O_9_ | 353.08804 [M-H]^-^ | 191.1; 179.0; 173.0; 135.0 | 355.10217 [M+H]^+^; 377.08395 [M+Na]^+^ | 163.0; 135.0 | 9.33 | 300; 326 | [1][2] |
| **Aesculetin** | C_9_H_6_O_4_ | 177.01996 [M-H]^-^ | 149.0; 133.0; 105.0 | 179.03399 [M+H]^+^ | 151.0; 133.0; 123.0 | 9.61 | - | [4] |
| **Chlorogenic acid isomer (isochlorogenic acid A, B or C)** | C_16_H_18_O_9_ | 353.08804 [M-H]^-^ | 191.1; 179.0; 173.0; 135.0 | 355.10217 [M+H]^+^; 377.08395 [M+Na]^+^ | 163.0; 135.0 | 9.88 | - | [1][2] |
| **Fraxetin** | C_10_H_8_O_5_ | 207.03047 [M-H]^-^ | 192 | 209.04435 [M+H]^+^; 231.02634 [M+Na]^+^ | 209.0; 194.0; 181.0; 163.0; 153.0; 149.0; 135.0 | 10.19 | - | - |
| **Coumaric acid hexoside isomer** | C_15_H_18_O_8_ | 325.09327 [M-H]^-^; 371.09916 [M-H+FA]^-^ | 163.0; 119.0 | 327.10744 [M+H]^+^; 344.13401 [M+NH_4_]^+^; 349.08935 [M+Na]^+^ | 165.1; 147.0 | 10.32 | 276; 313 | - |
| **Coumaric acid hexoside isomer** | C_15_H_18_O_8_ | 325.09327 [M-H]^-^; 371.09916 [M-H+FA]^-^ | 163.0; 119.0 | 327.10744 [M+H]^+^; 344.13401 [M+NH_4_]^+^; 349.08935 [M+Na]^+^ | - | 10.76 | - | - |
| **Coumarin sulfate with 2 methoxy moieties (iso-fraxidin or fraxidin)** | C_11_H_10_O_8_S | 301.00267 [M-H]^-^ | 221.0; 206.0; 191.0; 163.0 | 303.01702 [M+H]^+^ | 223.1; 208.0; 190.0; 135.0 | 11.54 | 292; 334 | - |
| **Coumarin sulfate (fraxetin-*O*-sulfate isomer)** | C_10_H_8_O_8_S | 286.98758 [M-H]^-^ | 207.0; 192.0 | 289.00137 [M+H]^+^ | 209.0; 194.0; 181.0; 163.0; 153.0; 149.0; 135.0 | 11.65 | 296; 342 | - |
| **Not identified** | C_12_H_18_O_7_S | 305.07022 [M-H]^-^ | 225.1; 181.1; 147.1; 97.0 | - | - | 11.74 | - | - |
| **Coumarin sulfate (fraxetin-*O*-sulfate isomer)** | C_10_H_8_O_8_S | 286.98758 [M-H]^-^ | 207.0; 192.0 | 289.00137 [M+H]^+^ | 209.0; 194.0; 181.0; 163.0; 153.0; 149.0; 135.0 | 11.78 | 300; 341 | - |
| **Scopoletin** | C_10_H_8_O_4_ | 191.03563 [M-H]^-^ | 176.0; 148.0; 104.0 | 193.04921 [M+H]^+^ | 178.0; 133.0 | 12.03 | 301; 336 | [4] |
| **Coumarin with 2 methoxy moieties (iso-fraxidin or fraxidin)** | C_11_H_10_O_5_ | 221.04623 [M-H]^-^ | 206.0; 191.0; 163.0 | 223.06034 [M+H]^+^; 245.04239 [M+Na]^+^ | 208.0; 190.0; 135.0 | 12.11 | 300; 341 | [4] |
| **Coumarin sulfate (scopoletin-*O*-sulfate isomer)** | C_10_H_8_O_7_S | 270.99296 [M-H]^-^ | 191.0; 176.0; 148.0 | 273.00636 [M+H]^+^ | 193.0; 178.0; 165.0; 149.1; 133.0 | 12.19 | 282; 339 | - |
| **Fraxidin-caffeoyl-hexoside** | C_26_H_26_O_13_ | 545.13217 [M-H]^-^ | 323.1; 221.0; 206.0; 191.0; 179.0; 163.0; 135.0 | 547.14421 [M+H]^+^; 564.17128 [M+NH_4_]^+^; 569.12661 [M+Na]^+^ | 325.1; 223.1; 208.0; 190.0; 181.0; 135.0 | 13.03 | 297; 328 | - |
| **Methoxy-cinnamic acid** | C_10_H_10_O_3_ | 177.05635 [M-H]^-^ | 162.0; 134.0 | 179.07054 [M+H]^+^ | 161.1; 147.0; 133.1; 119.0; 105.1; 91.0 | 13.53 | - | [2][5] |
| **Dicaffeoylquinic acid** | C_25_H_24_O_12_ | 515.11950 [M-H]^-^ | 353.1; 191.1; 179.0; 173.0; 161.0; 155.0; 135.0 | 517.13405 [M+H]^+^ | 163.0; 145.0; 135.0; 117.0 | 13.65 | 300; 325 | [4] |
| **Dicaffeoylquinic acid** | C_25_H_24_O_12_ | 515.11950 [M-H]^-^ | 353.1; 191.1; 179.0; 161.0; 155.0; 135.0 | 517.13405 [M+H]^+^ | 163.0; 145.0; 135.0; 117.0 | 14.06 | 300; 328 | [4] |
| **Dicaffeoylquinic acid methyl ester** | C_26_H_26_O_12_ | 529.13515 [M-H]^-^ | - | 531.14914 [M+H]^+^ | - | 14.35 | - | - |
| **Dicaffeoylquinic acid** | C_25_H_24_O_12_ | 515.11950 [M-H]^-^ | 353.1; 191.1; 179.0; 173.0; 161.0; 155.0; 135.0 | 517.13405 [M+H]^+^ | 163.0; 145.0; 135.0; 117.0 | 14.45 | 300; 328 | [4] |
| **Dicaffeoylquinic acid methyl ester** | C_26_H_26_O_12_ | 529.13515 [M-H]^-^ | 367.1; 353.1; 191.1; 179.0; 173.0; 161.0; 135.0 | 531.14914 [M+H]^+^ | 177.0; 163.0; 145.0; 135.0; 117.0 | 14.6 | - | - |
| **Dicaffeoylquinic acid** | C_25_H_24_O_12_ | 515.11950 [M-H]^-^ | 353.1; 191.1; 179.0; 173.0; 161.0; 155.0; 135.0 | 517.13405 [M+H]^+^ | 163.0; 145.0; 135.0; 117.0 | 14.79 | 300; 325 | [4] |
| **Dicaffeoylquinic acid methyl ester** | C_26_H_26_O_12_ | 529.13515 [M-H]^-^ | 367.1; 353.1; 191.1; 179.0; 173.0; 161.0; 135.0 | 531.14914 [M+H]^+^ | 177.0; 163.0; 145.0; 135.0; 117.0 | 15.03 | - | - |
| **Dicaffeoylquinic acid methyl ester** | C_26_H_26_O_12_ | 529.13515 [M-H]^-^ | 367.1; 353.1; 191.1; 179.0; 173.0; 161.0; 135.0 | 531.14914 [M+H]^+^ | 177.0; 163.0; 145.0; 135.0; 117.0 | 15.37 | - | - |
| **Caffeic acid coupled to C_11_H_12_O_6_** | C_20_H_18_O_9_ | 401.08864 [M-H]^-^ | 239.1; 179.0; 149.1; 135.0 | 403.1027 [M+H]^+^; 420.12903 [M+NH_4_]^+^; 425.08439 [M+Na]^+^ | 163.0; 135.0 | 15.89 | 304; 325 | - |
| **Flavonoid** | C_15_H_12_O_6_ | 287.05684 [M-H]^-^ | 151.0; 135.0; 125.0; 107.0 | 289.07104 [M+H]^+^ | 179.0; 163.0; 153.0; 135.0 | 15.99 | 286 | - |
| **Ethoxy or Dimethoxycinnamic acid** | C_11_H_12_O_4_ | 207.06657 [M-H]^-^ | 179.0; 161.0; 135.0; 133.0 | 209.0811 [M+H]^+^ | 181.0; 163.0; 145.0; 135.0; 117.0 | 16.1 | 295; 325 | - |
| **Tricaffeoylquinic acid** | C_34_H_30_O_15_ | 677.15139 [M-H]^-^ | 515.1; 353.1; 335.1; 191.1; 179.0; 173.0; 161.0; 155.0; 135.0 | 679.16635 [M+H]^+^; 696.19325 [M+NH_4_]^+^; 701.14839 [M+Na]^+^ | 499.1; 163.0; 145.0; 135.0 | 16.13 | 295; 329 | - |
| **Dimethoxyflavonoid (axillarin)** | C_17_H_14_O_8_ | 345.0621 [M-H]^-^ | 330.0; 315.0; 287.0; 259.0; 149.0 | 347.07648 [M+H]^+^ | 332.0; 317.0; 289.0; 261.0; 186.0; 168.0 | 16.41 | 271; 349 | - |
| **Methoxyflavonoid (tamarixetin, rhamnetin, eupafolin, quercetin-3-methylether)** | C_16_H_12_O_7_ | 315.05118 [M-H]^-^ | 300.0; 228.0 | 317.0661 [M+H]^+^ | 302.0; 168.0 | 16.72 | 272; 345 | [4] |
| **Methoxyflavonoid (laricitrin or mearnsetin)** | C_16_H_12_O_8_ | 331.04646 [M-H]^-^ | 316.0; 287.0; 271.0; 243.0; 209.0; 181.0; 166.1 | 333.06097 [M+H]^+^ | 318.0; 290.0; 244.0 | 16.8 | - | - |
| **Trimethoxyflavonoid** | C_18_H_16_O_8_ | 359.07785 [M-H]^-^ | 344.0; 329.0; 314.0; 301.0; 286.0; 258.0; 242.0; 214.0 | 361.09201 [M+H]^+^ | 346.1; 330.0; 317.1; 300.1; 285.0; 275.0; 257.0; 229.0; 187.0; 169.0 | 17.49 | - | - |
| **Dimethoxyflavonoid (cirsiliol)** | C_17_H_14_O_7_ | 329.06699 [M-H]^-^ | 314.0; 299.0; 271.0; 255.0; 227.0; 199.0 | 331.08188 [M+H]^+^ | 316.1; 301.0; 273.0; 245.0; 186.0; 168.0 | 17.97 | 273; 345 | - |
| **Trimethoxyflavonoid** | C_18_H_16_O_8_ | 359.07785 [M-H]^-^ | 344.0; 329.0; 314.0; 301.0; 286.0; 258.0; 242.0; 214.0 | 361.09201 [M+H]^+^ | 346.1; 330.0; 317.1; 300.1; 285.0; 275.0; 257.0; 229.0; 187.0; 169.0 | 18.13 | 275; 329 | - |
| **Methoxyflavonoid (hispidulin)** | C_16_H_12_O_6_ | 299.05618 [M-H]^-^ | 284 | 301.07092 [M+H]^+^ | 286.0; 168.0 | 18.26 | 273; 329 | [6] |
| **Trimethoxyflavonoid (cirsilineol or eupatorin)** | C_18_H_16_O_7_ | 343.08286 [M-H]^-^ | 328.1; 313.0; 298.0; 285.0; 270.0; 242.0; 163.0 | 345.09719 [M+H]^+^ | 330.1; 315.0; 287.0; 169.0 | 18.89 | 272; 347 | [4] |
| **Tetramethoxyflavonoid** | C_19_H_18_O_8_ | 373.09376 [M-H]^-^ | 358.1; 343.0; 328.0; 313.0; 300.0; 285.0; 257.0; 241.0; 177.1; 163.0 | 375.1073 [M+H]^+^ | 360.1; 327.0; 314.1; 299.1; 285.0; 271.1; 257.0; 243.1; 229.0; 169.0 | 19.25 | 281; 327 | - |
| **Trimethoxyflavonoid (cirsilineol or eupatorin)** | C_18_H_16_O_7_ | 343.08286 [M-H]^-^ | 328.1; 313.0; 299.1; 285.0; 270.0; 242.0; 163.0 | 345.09719 [M+H]^+^ | 329.1; 315.0; 284.1; 255.1 | 19.48 | - | [4] |
| **Dimethoxyflavonoid (cirsimaritin)** | C_17_H_14_O_6_ | 313.07203 [M-H]^-^ | 298.0; 283.0; 255.0; 227.0; 163.0 | 315.08657 [M+H]^+^ | 300.1; 285.0; 168.0; 135.0 | 20.07 | 272; 335 | [4] |
| **Linderoflavone B** | C_20_H_18_O_8_ | 385.09385 [M-H]^-^ | - | 387.10797 [M+H]^+^ | - | 21.2 | - | [4] |

*RT – retention times

**References**

[1] Sebai, H., Jabri, M.-A., Souli, A., Hosni, K., Selmi, S., Tounsi, H., Tebourbi, O., Boubaker, S., El-Benna, J., Sakly, M. Protective effect of *Artemisia campestris* extract against aspirin-induced gastric lesions and oxidative stress in rat. *RSC Adv.* **4,** 49831-49841 (2014).

[2] Dib, I., Angenot, L., Mihamou, A., Ziyyat (Pr), A., Tits, M. *Artemisia campestris* L.: Ethnomedicinal, phytochemical and pharmacological review. *J. Herb. Med.* **7,** 1-10 (2016).

[3] González, A.G., Bermejo, J., Estévez, F.,  Velázquez, R. Phenolic derivatives from Artemisia glutinosa. *Phytochemistry* **22,** 1515-1516 (1983).

[4] Megdiche-Ksouri, W., Trabelsi, N., Mkadmini, K., Bourgou, S., Noumi, A., Snoussi, M., Barbria, R., Tebourbi, O., Ksouri, R. *Artemisia campestris* phenolic compounds have antioxidant and antimicrobial activity. *Ind. Crops Prod.* **63,** 104-113 (2015).

[5] Riedel, H., Cai, Z., Smetanska, I. Obtaining phenolic acids from cell cultures of various Artemisia species. *Afr. J. Biotechnol.* **9,** 8805-8809 (2010).

[6] Hurabielle, M., Eberle, J., Paris, M. Etude des flavonoïdes d'Artemisia campestris sous-espèce Glutinosa [Flavonoids of Artemisia campestris, ssp glutinosa]. *Planta Med.* **46,** 124-125 (1982).
